# Functional features in patients with idiopathic macular hole treatment via OCT angiography

**DOI:** 10.1097/MD.0000000000031862

**Published:** 2022-11-25

**Authors:** Jing Li, Wenjuan Wang, Bin Sun, Xiaodan Zhang, Tong Cui, Peini Cheng, Zhijie Jia, Jingjing Wang, Guohong Zhou

**Affiliations:** a Department of Ophthalmology, Shanxi Eye Hospital, Taiyuan, Shanxi, China; b Department of School of the 1st Clinical Medical Sciences, Shanxi Medical University, Taiyuan, Shanxi, China.

**Keywords:** idiopathic macular hole, OCT-angiography, pars plana vitrectomy

## Abstract

To evaluate the optical coherence tomography (OCT) angiography features in patients with idiopathic macular hole (IMH) before and after vitrectomy.

This prospective study included 25 patients diagnosed with IMH in Shanxi eye hospital from August 2019 to December 2021. The study was divided into 3 groups: IMH eyes, fellow eyes and normal eyes. All unilateral IMH eyes underwent vitrectomy.

There were significant differences in superficial retinal blood flow density (SRBFD, *P* < .001) and choroidal blood flow density (CBFD) between IMH and healthy control eyes before operation (*P* < .05). There was significant difference in SRBFD between fellow eyes and normal eyes (*P* = .038). The changes of SRBFD and CBFD in IMH eyes before and after operation were statistically significant (*P* < .05). The CBFD at 6 months after operation is negatively correlated with LogMAR visual acuity, and the CBFD of the fellow eye is also negatively correlated with LogMAR visual acuity. The SRBFD and CBFD had no correlation with the diameter of macular hole before and after operation.

SRBFD and CBFD increased after vitrectomy, indicating that the blood supply of retina and choroid were partially restored after vitrectomy. There was no correlation between SRBFD, CBFD and hole diameter, but there was correlation between choroidal blood flow and LogMAR visual acuity.

## 1. Introduction

Idiopathic macular hole (IMH) is the main cause of central vision loss in the elderly.^[1]^ Vitrectomy combined with internal limiting membrane (ILM) dissection has been identified as the main treatment for IMH.^[2,3]^ Although macular hole closure rates are higher after vitrectomy, but improvement in functional vision remains unsatisfactory. The macula is vascularized by a network of capillaries consisting of circulating chorionic capillaries. Some studies have suggested that anatomical changes in the choroid may be related to the development of retinal defects and the progression of macular holes.^[4]^ Most studies on IMH surgery have optimal corrected vision (BCVA) as the primary indicator of recovery.^[5]^ Although BCVA is an effective measurement in some cases, it does not fully reflect retinal function.

Previously, researchers speculated that a decrease in blood flow to the fovea choroid may be related to the formation of IMH. However, due to the limitations of traditional detection equipment, traditional coherence tomography (OCT) cannot quantitatively analyze capillary networks. In addition, in clinical practice, the detection of choroidal blood flow mainly uses indocyanine green angiography (ICGA).^[6]^ However, it requires an intravenography and is an invasive test, so it is not suitable for everyone. OCT Angiography (OCTA) is a newly established 3-dimensional OCT for the visualization and evaluation of retinal blood vessels. The application of OCTA enables noninvasive measurement of choroidal microvascular flow.^[7–9]^ However, little is known about changes in choroidal microvascular flow by OCTA in IMH eyes.

In this study, we investigated the superficial retinal blood flow densities and choroidal blood flow densities in the macula of IMH patients and compared them with the fellow eyes and normal eyes to determine IMH formation and prevention and Potential avenues for the treatment of IMH. At the same time, the correlation between blood flow density and macular hole diameter, blood flow density and LogMAR visual acuity was detected.

## 2. Methods

This was a prospective study and was approved by the Institutional Review Board of Shanxi Eye Hospital of Taiyuan, China (No. SXYYLL-20190802). The research protocol followed the principles of the Declaration of Helsinki (revised 2013). All patients signed informed consent before participating. The patient visited Shanxi Eye Hospital from August 2019 to December 2021.

All participants underwent visual acuity, refraction, non-contact intraocular pressure measurement, anterior segment examination and detailed fundus examination, OCT and OCTA. IMH staging according to Gass classification.^[8,10]^ According OCT, we get the size of IMH diameter. The test was performed by a spectral domain optical coherence tomography (SD-OCT) system (Optovue, Angiovue imaging system, American). All IMH eyes underwent 25G pars plana vitrectomy (PPV) plus ILM stripping plus gas (C3F8 or sterile air) or silicone oil implantation. Follow-up for 6 months. Best-corrected visual acuity (logMAR) in patients with IMH was tested before surgery, 1, 3, and 6 months after surgery. Healthy subjects over the age of 50 with a BCVA better than 0.9 and no eye disease were included in the healthy control group.

In order to quantify the blood circulation of the retina and choroid, the OCTA Sofware Version is AngioVue Analytics license. Optovue software (version 2014.2.0.93) was used to analyze the blood flow density of the superficial retinal angiography and the blood flow density of the deep choroidal layer, reflecting the choriocapillary circulation in the macular region. Divide this data by the area of the selected region 3.142 to obtain the blood flow density of the choroid layer. OCTA metrics were measured using a vascular retinal pattern scan (3 mm × 3 mm in size). The blood flow area of the superficial retinal capillaries in the macular area and the blood flow area of the choroidal capillaries are the blood flow area with the center of the macular fovea as the center, the radius is 1 mm, and the scanning range is 3 mm × 3 mm.

### 2.1. Statistical analysis

Data were entered into excel (Microsoft Corporation, Redmond, WA) and all analyses were performed in SPSS (version 23.0, Chicago, IL). Continuous variables were presented as mean ± standard deviation (SD), and categorical variables are presented as percentages. Normality of parameters was assessed with the Shapiro–Wilk test. Vessel density of superficial retinal capillaries and choroidal blood flow area was compared between IMH eyes and normal control eyes using independent samples *t* test. IMH eye data were compared between preoperative and postoperative 1, 3, and 6 months using repeated measures ANOVA. Paired samples *t* test was used for preoperative and postoperative analysis of variables. Spearman correlation were performed to investigate the correlation between visual acuity and superficial retinal blood flow density (SRBFD) and choroidal blood flow density (CBFD). The same method is used to analyze the correlation between the diameter of IMH and SRBFD and CBFD. *P* < .05 was considered statistical significant.

## 3. Results

In this study, 25 eyes of 25 patients with unilateral IMH were included, and 30 eyes of 30 healthy subjects were included as control. Six months after surgery, 16 eyes were closed in the U-shape (64%) and 9 eyes were closed in the V-shape (36%).

Table 1 shows the characteristics of the included eyes. There was no significant difference in age, gender and BCVA between IMH eyes, fellow eyes and normal eyes (all *P* > .05).

**Table 1:** Baseline characteristic of included patients.

	Patients		Healthy control (n = 30)	*P*
	IMH eyes (n = 25)	Fellow eyes (n = 30)		
Gender (male/female)	3/22	3/22	5/25	.502
Age (yrs)	63.80 ± 4	63.80 ± 4.30	61.00 ± 5.80	.069
BCVA (LogMAR)	1.65 ± 1.19	0.08 ± 0.05	0.06 ± 0.05	<.001
Duration (days)	86.20 ± 45.36			
Macular hole diameter (μm)	608.35 ± 4.30			

IMH = idiopathic macular hole, LogMAR = logarithm of the minimum angle of resolution.

The CBFD between the IMH eye group and the fellow eye group was statistically significant (*P* < .001). The SRBFD and CBFD between the IMH group and the normal control group were also statistically significant (*P* < .001 and *P* = .002, respectively). The SRBFD level in the same eye group was lower than that in the normal control group (*P* = .038, Table 2).

**Table 2:** Comparisons of SRBFD and CBFD measurements in 3 groups.

Region	IMH eyes (N = 25)	Fellow eyes (N = 25)	Normal eyes (N = 30)	*P*	*P*-value for IMH eyes vs. Fellow eyes	*P*-value for IMH eyes vs. normal eyes	*P*-value for Fellow eyes vs. normal eyes
SRBFD (mm^2^)	45.79 ± 3.49	48.20 ± 3.90	49.75 ± 3.33	<.001	.562	<.001	.038
CBFD (mm^2^)	0.44 ± 0.07	0.56 ± 0.07	0.59 ± 0.01	<.001	<.001	.002	<.001

CBFD = choroidal blood flow density, IMH = idiopathic macular hole, SRBFD = superficial retinal blood flow density.

Table 3 showed the differences between SRBFD and CBFD before and after surgery (1, 3, and 6 months). SRBFD was statistically significant at baseline, 1, 3, and 6 months postoperatively, and postoperative SRBFD increased (1 and 3 months). Likewise, the CBFD changes before surgery, 1, 3, and 6 months after surgery were also statistically significant.

**Table 3:** Pre and post-surgery comparison of SRBFD and CBFD in eyes with IMH (Mean ± SD).

Region	Blood flow density				*P*
	Pre-operation	1 mo	3 mo	6 mo	
SRBFD (mm^2^)	45.19 ± 0.64	46.35 ± 0.95	47.91 ± 0.75	43.56 ± 0.98	<.001
CBFD (mm^2^)	0.44 ± 0.08	0.49 ± 0.13	0.51 ± 0.14	0.53 ± 0.15	.002

BFD = blood flow density, CBFD = choroidal blood flow density, IMH = idiopathic macular hole, SRBFD = superficial retinal blood flow density.

Table 4 Correlation analysis between visual acuity and SRBFD, CBFD in the fellow eye and before and after operation of IMH eyes. We can see that the CBFD at 6 months after operation is negatively correlated with LogMAR visual acuity, and the CBFD of the fellow eye is also negatively correlated with LogMAR visual acuity.

**Table 4:** Correlation between visual acuity in logMAR and SRBFD, CBFD before and after surgery in IMH eyes and in the fellow eyes.

Region	IMH Eye Pre surgery		IMH Eye Post surgery (6 mo)		Fellow Eye	
	*R* value	*P* value	*R* value	*P* value	*R* value	*P* value
SRBFD (mm^2^)	−0.016	.948	−0.270	.278	0.159	.528
CBFD (mm^2^)	−0.145	.580	−0.482	.050	−0.499	.042

BFD = blood flow density, CBFD = choroidal blood flow density, IMH = idiopathic macular hole, SRBFD = superficial retinal blood flow density.

Table 5 The SRBFD and CBFD had no correlation with the diameter of macular hole before and after operation.

**Table 5:** Correlation analysis of macular hole size with retinal blood flow density and choroidal blood flow density.

	Pre surgery		Study Eye Post surgery (1 mo)		Study Eye Post surgery (3 mo)		Study Eye Post surgery (6 mo)	
	*R* value	*P* value	*R* value	*P* value	*R* value	*P* value	*R* value	*P* value
SRBFD (mm^2^)	0.318	.172	0.036	.884	−0.064	.789	0.032	.896
CBFD (mm^2^)	0.088	.729	0.137	.588	−0.027	.914	−0.221	.378

## 4. Discussion

**Figure 1.**
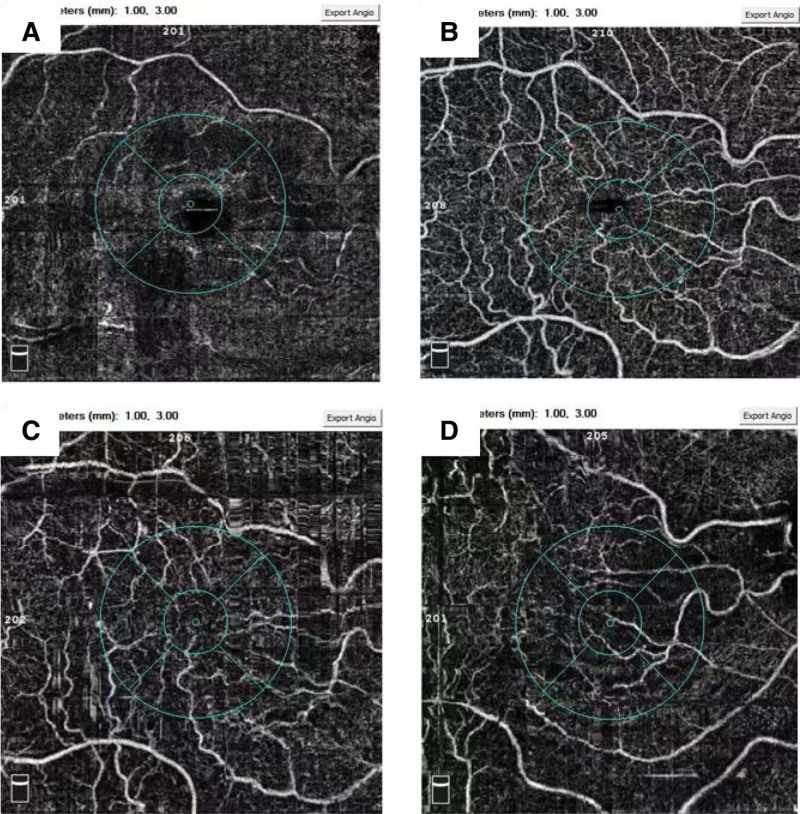
The SRBFD: It can be seen that the blood flow density of the superficial retina has increased after 1 and 3 months compared with pre-operation. SRBFD = superficial retinal blood flow density.

**Figure 2.**
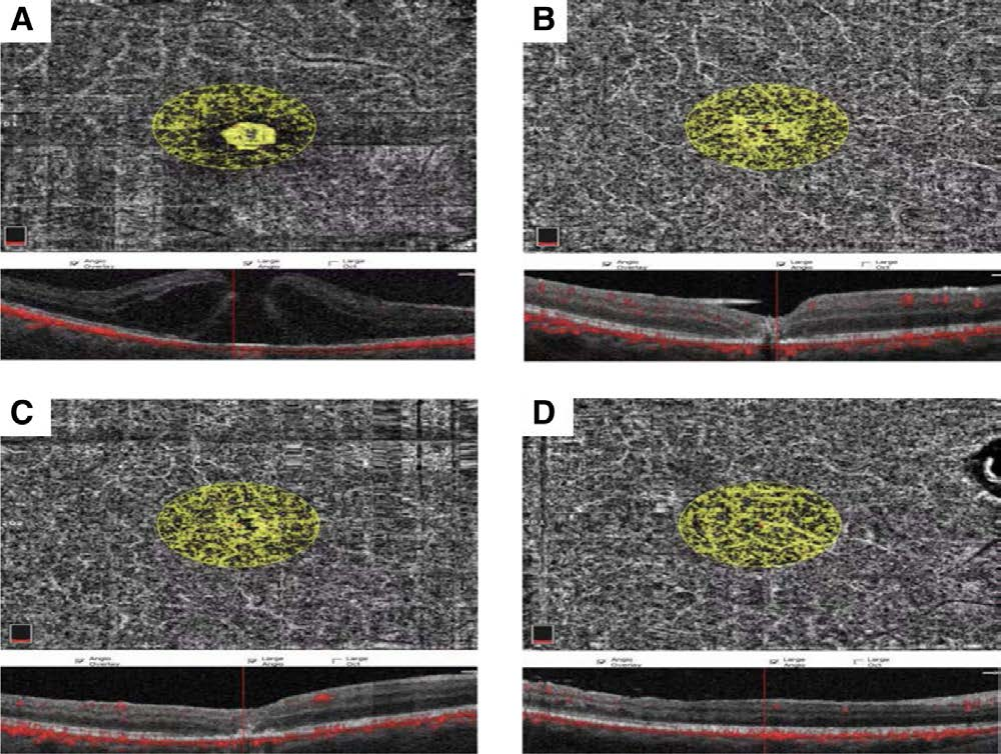
The choroidal blood flow area: It can be seen that 1, 3 and 6 months after the operation, the choroidal blood flow area gradually increased compared with pre-operation.

This study found that after macular hole surgery, the macular hole was successfully closed, and the macular function was improved. We verified the morphology and function of retina and choroid, and also observed the correlation between retinal and CBFD and macular hole diameter and visual acuity. In OCTA measurement, the levels of SRBFD and CBFD after operation were higher than those before operation, and the SRBFD and CBFD of macular hole were significantly lower than those of normal eyes. Therefore, we should be alert to the occurrence of macular hole in the fellow eye, and do regular follow-up, early detection and early intervention in the follow-up work. At the same time, we can see that the CBFD at 6 months after operation is negatively correlated with LogMAR visual acuity, and the CBFD of the fellow eye is also negatively correlated with LogMAR visual acuity. At the same time, the choroidal blood flow in the fellow eyes was also correlated with visual acuity, indicating that the CBFD was closely related to visual acuity to a certain extent. The choroidal blood flow was restored and the visual acuity was also improved after operation in 6 months, indicating that the choroidal blood supply was restored, and the patient’s visual function was also improved;

The choroid is a source of nutrients for the macular area and is the blood supply tissue of the macular area. Choroidal capillary injury seriously affects the function of the macular area and the effect on vision is fatal. Therefore, it is very important to accurately understand the blood circulation of the choroid. The occurrence, development and prognosis of macular hole are closely related to changes in blood circulation. So we can measure blood circulation in the choroid to assess disease progression and treatment outcomes.^[6]^ Due to the lack of technical means to measure choroidal blood flow in the past, we cannot know the effect of choroidal blood flow on the pathogenesis of macular hole, and the pathogenesis of IMH mainly focuses on the study of choroidal thickness. Traditionally, due to the different quantitative methods of choroidal thickness measurement, the results of various studies are controversial and there are different theories.^[7,11,12]^ Gao Y et al showed the decrease of choroid thickness may occur before the macular hole formation. It was verified again that the choroidal blood flow area in macular fovea of IMH patients was significantly lower than that in fellow eyes and healthy eyes.^[13]^With the advent of OCTA,^[14]^ Quantitative analysis of retinal and CBFD became possible. There have been few studies of retinal and choroidal circulation in the macular area before and after IMH surgery. Most of the previous studies have focused on the morphology of the retina and choroid before and after macular foramen surgery, such as the change of the area of superficial vascular area of the macula and the change of choroid thickness.^[8,10,15]^ Zhou N et al findings suggested that atrophy of choriocapillary might play an important role in the formation of IMH.^[16]^ In our study, SRBFD increased at 1 and 3 months after surgery, indicating that retinal function was partially restored, surgery could repair the morphology of IMH, but whether the function of the macular retina could be restored to its original state, our study showed that with the closure of the macular hole, the function of the macular area is also gradually restored, local blood flow metabolism is gradually restored, so choroidal capillary perfusion is required to provide the required nutritional supply, CBFA increased significantly, That is, to compensate for the nutrients needed for the retinal tissue to restore function. Compared with the fellow eyes, The CBFD of IMH eyes was significantly reduced and increased at different postoperative periods (1, 3, and 6 months). These changes reflect the tendency of retinal structure to return to normal after IMH surgery with the closure of the macular hole, corresponding to the need to increase choroidal blood supply to maintain the function of the retina.^[12]^ We can clearly see that the SRBFD and choroidal blood flow area before operation were both lower than normal eyes, and were improved during the follow-up period after surgery from Figures 1 and 2.

There are also some shortcomings in this study, including a small sample size, a short observation time, and some limitations in the results of the study. It is necessary to expand the sample and conduct in-depth research. Our follow-up period is 6 months, and it is necessary to conduct further long-term observations and continue to study changes in retinal and choroidal function to provide more theoretical basis.

## 5. Conclusion

According to OCTA, SRBFD and CBFD were significantly lower in IMH eyes than in healthy eyes. SRBFD increased at 1 and 3 months postoperatively, and postoperative CBFD continued to increase. Our study showed that retinal function and choroidal circulation can be partially restored in IMH eyes after macular hole closure, and quantification of SRBFD and CBFD can be used as parameters to assess macular disease progression and prognosis. At the same time, we also found that the CBFD was correlated with visual acuity at 6 months after operation; the retinal and CBFD had no correlation with the diameter of macular hole.

## Author contributions

GHZ conceived and designed the study. JL, WJW, BS, PNC and XDZ collected and reviewed the patient data. TC, JJW and ZJJ analyzed and interpreted the data. JL was a major contributor to manuscript writing. All authors have read and approved the final manuscript.

**Data curation:** Jing Li, Wenjuan Wang, Bin Sun, Xiaodan Zhang, Tong Cui, Peini Cheng, Zhijie Jia, Jingjing Wang, Guohong Zhou.

**Formal analysis:** Tong Cui, Guohong Zhou.

**Funding acquisition:** Zhijie Jia, Jingjing Wang.

**Methodology:** Jing Li, Jingjing Wang.

**Project administration:** Jing Li.

**Writing – original draft:** Jing Li.

**Writing – review & editing:** Bin Sun, Guohong Zhou.
